# Translations

**Published:** 1848-01-01

**Authors:** 

**Affiliations:** ASSISTANT-PHYSICIAN TO THE LUNATIC ASYLUM AT EBERBACH


### Translations.

### IS THE PENNSYLVANIAN OR CELL-SYSTEM FAVOURABLE TO THE DEVELOPMENT OF INSANITY?

(*From the German.*)

BY DR. BASTING,

ASSISTANT-PHYSICIAN TO THE LUNATIC ASYLUM AT EBERBACH.*

M. APPERT, from Paris, has attempted, in a recent publication, (Travels in Prussia,) to direct the attention of the public to the silent system, as adopted at Berlin, in the new prison for the punishment of criminals. He observes, "I felt melancholy when I entered this prison on the 13th of September. This bee-hive, this dead-house, whence the prisoner, afar from the other world, never, living or dead, departs; where the captive can never once taste the innocent enjoyment of the green fields. My head was troubled to think that so much money had been expended on this receptacle of misery and curses, whose last stone is scarcely placed ere its foundation begins to shake beneath the repeated strokes of a unanimous disapproval on the part of science, religion, humanity, and justice."

Similar declamations have been found of late years in the public journals, written for the purpose of prejudicing the public against a system of punishment, the precedents of which have been demonstrated in North America, England, France, and Switzerland; imperfectly indeed, as regards the construction of the cells, and the management of the prisoners, or so far as their superintendence, employment, and care and attention to their mental and bodily health are concerned. These public declamations may serve to lead the inexperienced and even governments into erroneous views. What do they contain worthy of notice, if they are altogether groundless? Since it cannot be denied that a judicious application of the cell-system is the only means in the power of the state for the bettering the condition of felons, it seems much more reasonable to facilitate the introduction of the system by bringing forward established facts, than to oppose it by probable suppositions, and to endeavour to find out the means and modes whereby the partly groundless objections to the cell-system may be met.

M. Appert is for solitary confinement during the night, but decides against total isolation. "No judge has the right," he says, "to doom a human being to insanity," only it is yet to be demonstrated, psychologically, that the Pennsylvanian system has produced such results in a single individual.

A cell which contains at the least 1000 cubic feet, with freedom of motion assured to the prisoner, dry, well ventilated, well lighted, warmed

* Read at the Section for Psychiatry, of the Association of German Naturalists, held at Brunswick, 1846.

according to the season, in which the prisoner has a warm bed, sufficient and wholesome food, suitable clothing, and proper employments—in which his bodily and mental welfare are watched over by the superintendent physician, chaplain, officers, and turnkeys, and when exercise in the open air for at least half an hour daily is permitted, such a cell cannot possibly favour the development of insanity, but rather, on the contrary, would be prophylactic.

Many crimes are committed during a period of moral irresponsibility. The mad-houses of all countries have no small proportion of their inmates who were first received after they had given warning of their dangerous condition by some overt act, or whose insanity was first ascertained during a criminal investigation. With how many criminals has it happened, that the criminal act was committed in a paroxysm of insanity, but that during the investigation they appeared to be of sound mind. How many an insane person has been freed from his malady by some sudden influence on the mind. How many embarrassments do not the advocates of the accused present to judges, and physicians when they attempt to represent their clients as irresponsible agents. Celebrated physicians of lunatic hospitals have had regard to a special arrangement of the buildings entrusted to their superintendence, in which they could separate from the rest, and treat the criminals placed under their care by judges, for the purpose of determining their mental condition. I have known many instances in which such criminals have become actually insane, and have been transferred to the hospital for treatment. Suicides are very frequent amongst suspected criminals, and many are committed during a period of mental aberration. According to a paragraph, which appeared in the Frankfort Journal for Dec. 11th, 1845, within the period of one year, twenty persons had hung themselves in the "Inquisitoriate" at Glatz. Besides the loss of freedom, there are other active agencies in operation likely to overthrow the mental equilibrium, as loss of honour, shame, a reproving conscience, anxiety and fear of punishment; probably, in many instances, to these may be added defective superintendence, and the annoyance of incessant cross-examinations. I know educated persons who have been cured of insanity who have given me instructive explanations of the partially insane condition, and who pitied every prisoner; declaring that to them it appeared extremely probable that the greater number of crimes and of immoral actions were perpetrated under a powerful insane impulse against which the soul struggled. All this leads to the conclusion, that more or less insanity is frequent amongst criminals; that the greater number of crimes are committed in a state of the system not excluding moral responsibility, but in which it is indeterminate and obscure, and which may be easily changed into insanity; and, consequently, that every prison is liable to have insanity developed in it.

But that the educated man who, whether from passion or other motive, has committed a crime—who finds himself surrounded in prison by hardened and dissolute men of every kind—who has no sympathy with the rudeness of the abandoned—to whom it must be tormenting to find himself in such society—who feels the want of his accustomed comforts and friends—pursued, perhaps, by a desire for dear relatives, or by bitter remorse—probably finding himself sneered at and despised, mocked and maltreated for his better feelings—that such a pitiable person is more likely to become insane amongst a crew of reprobates than in an airy wholesome cell, and supplied with all the attention necessary to his bodily and mental welfare, is to me a certainty. Time and opportunity is afforded to him in his cell to converse with the chaplain, to quiet his conscience, to form good resolutions, to seek consolation in undisturbed meditation and prayer, to amuse himself with labour. And if his soul is burdened with the weight of his transgressions, with the reproaches of his relatives, and despair presses upon him even to mastery, where will he the sooner find peace—in the society of felons, or in the calm of his own abode? It is possible that amongst the vicious he will become vicious; that he may take a part in their dissipation, their conspiracies, and war against society, and his conscience may thus be benumbed; but the benumbed and not soothed conscience will at last awake in him, and must excite a fearful struggle in his soul, amid which his senses may be easily disordered.

The desire for freedom and for society is innate in us. The man with unburdened conscience, happy, and enjoying life, feels a happiness in the enjoyment of his freedom; he finds repose, amusement, and pleasure from companionship with those who are like-minded with himself, from honourable strife, from intellectual men. He knows how to use his freedom aright; he disturbs not social life; he endeavours to exalt its excitement, and finds only pleasure in the enjoyment of his existence when he can spend it amongst good men. He only half enjoys his pleasure when he cannot communicate it; there is nothing so painful to him as loneliness, or seclusion from his fellows.

Is this so with the criminal? Has he not before his imprisonment fled the light of day, and chosen the night for the exercise of his wickedness? What was more burdensome to him than the society of good men? What sought he to hide more carefully than himself and his crimes? To him the imprisonment affords an opportunity to live entirely according to his inclinations; he teaches and learns wickedness in his companionship; he no longer sighs for anything except freedom, that he may apply all he has learnt to his criminal vocation. He now goes more skilfully to work; he is more perfect in dissimulation, more perfect in the art of escaping justice; he made friends who help him in his enterprizes.

But if the desire for freedom be innate, so also is the desire for solitude. Who has not felt its kindly influence when he has had to bear real or imaginary evils? Where do we most freely pour forth our tears when death has torn a dear relation from us, when we have to endure persecution, when we have to lament our adverse fate? Do we not seek solitude when we would examine our heart and our actions? Do we not find in solitude the means of calming our conscience, of forming good resolutions? Does not our Saviour Jesus Christ himself teach us?—"But when thou prayest, enter into thy closet, and when thou hast shut thy door, pray to thy Father which is in secret, and thy Father which seeth in secret shall reward thee openly." To whom, then, is solitude more necessary than to the fallen sinner, to the repentant criminal?—and repentant we would have every criminal to become. To whom is prayer more necessary than to him? and we must afford him the opportunity of praying—it is our duty. But can we do this better in the society of hardened felons or in solitude? Can the man who has been led into crime either by the seduction of others, or by his own viciousness or indolence, and paying the penalty of his fault, return to rectitude, form good resolutions, and, in short, become a better man, in the same element that led him to crime? What a crying wrong is presented to his mind when he considers with himself—I am punished for crime, and brought into society where crime is held to be honourable, where the worst man is the most respected, the most contented, the most envied; I am brought into society where the best man is despised, scoffed at, degraded, insulted, abused; I am brought into society where every good resolution is ridiculed and accounted as madness, where in slang language the laws and institutions of society are defamed, and looked upon as hindrances which the bold youth must oppose with craft and cunning.

Let those who stigmatize the Pennsylvanian system of punishment as a disgrace to humanity, remember that one-third of the unfortunate people who harbour in the houses of correction have relapsed into crime, and that if they had taken the trouble to inquire of the criminals they would have learned that they were become habitual felons. Let them inquire, and they will hear that these persons have learned in the prisons to shun virtue, piety, and industry. Let them ask of the prison chaplains the reasons why so few prisoners are improved—why so few return to the paths of virtue—why almost all love vice and crime? Those who have to fulfil their difficult duty, with a perseverance and patience not sufficiently appreciated, will answer, as they have answered to me—The endeavour to improve the morals of the prisoners, so long as one corrupts another, is (to use their own expression) like thrashing empty straw.

The opponents of the Pennsylvanian system cannot get over this irrefragable truth; and so long as they can bring no better means to meet these acknowledged evils of prisons than the silent system, the impracticability of which has been fully demonstrated, they should earnestly suggest means which, in the opinions of those conversant with the matter, *can* meet those evils, before they venture to condemn it.

Amongst the principal objections to the cell system is the assertion that it favours the development of insanity. For the illustration of this I beg leave to investigate the extent to which insanity occurs amongst prisoners generally, and specially amongst those subject to solitary confinement, and with particular reference to facts stated on the authority of physicians and officers of prisons.

In every prison it may be asserted that at least five per cent. of the population is in a doubtful condition as to soundness of mind. It is the opinion of Blut and Baillarger that mental disorders are from four to six times more prevalent amongst prisoners than a general population. The latter founds his opinions on the following irrefragably true principles:—

1. There are insane persons whose disease appears before and after sentence, and is first ascertained after entering the house of correction, in consequence of a medical investigation.

2. Many crimes are committed during the period of incipient insanity by such persons who are not quite insane, but who became so after condemnation.

3. Amongst prisoners there is a certain number whose whole general habit (*persönlichkiel*) seems to be the result of commencing disease, and which in a higher degree predisposes to every form of mental derangement.

4. Prisoners, both before and after condemnation, are exposed to many causes of mental disorder.

5. Simulated madness is more frequent in the penitentiaries, and it is easily comprehended how in this way errors may have arisen.

6. The discipline of prisons may become a cause of insanity amongst the inmates.

If persons would take the trouble to compare the number of cases of insanity that have occurred in ordinary prisons hitherto with those which are found in well-constructed cell prisons, the comparison would doubtless be much in favour of solitary confinement. In consequence of the greater attention with which the Pennsylvanian system has been watched, the frequent occurrence of insanity must strike the attention; whereas this has not happened as regards houses of correction, because they have not been so carefully observed. The third proposition of Baillarger is, however, quite true. Very frequently a certain number of prisoners are found with a partial aberration of mind. They are generally either those who have been imbecile from youth, or those in whom education has been neglected or entirely wanting, or those in whom, in consequence of a dissolute life, the mind has been remarkably weakened. Vice and insanity must, as Koller has established, be carefully distinguished from each other: there are cases, nevertheless, in which it is difficult to mark the point of transition from the one to the other, however easy this may appear to the uninitiated. Also, that insanity is more frequent amongst prisoners from the educated classes than the uneducated, is dependent upon the fact that the change of circumstances more deeply affects them; and this is not remarkable, when it is considered that they are more shocked by their captive condition, more restrained from their ordinary pursuits, and that the life of a prisoner corresponds but little to their early life, which with the uneducated is not the case. High official persons and officers of rank enjoy the privilege of expiating their offences in fortresses,—there is little restraint on their freedom, or limitation as to their diet and clothing; nevertheless, confinement has a depressing effect on both mind and body. In fact, restriction of freedom, of habits, and of modes of life, acts injuriously on the body and mind, and predisposes to corporeal and mental disorders. Wherefore, then, must the prisoner of a well-furnished cell be specially subject to these disorders? Experience teaches to the contrary, and also the opinions of highly learned and experienced men, who, as physicians, have attained to an indisputable reputation.

A commission appointed by the Royal Academy of Paris, and consisting of Esquirol, Marc, Villerue, Louis, and Pariset, in reply to the French government, gave it, on Jan. 3rd, 1839, as their opinion, publicly and positively expressed, that the fear of injury to the mental health of prisoners from solitary confinement was groundless.

Flemming, also, of Sachsenberg, near Schwerin, has enunciated the same opinion, for which he assigns the following grounds:—

1. It is quite correct that to men withdrawn from social relations it is an absolute requisite that they be, not only not left alone with their own thoughts, but that they be able to communicate them,—a requisite that they be understood,—a requisite, in short, that they be stimulated by the communication of the ideas of another, and that the absolute deprivation of this communication and stimulus may develope imbecility. Such absolute deprivation is, however, neither conceivable nor possible. No one can say that the Auburn system excludes this danger, inasmuch as the silent system, fully carried out, must have similar results. The Pennsylvanian system is, however, widely remote from requiring absolute solitariness, and absolute deprivation of the opportunity to receive and communicate ideas; it restrains communication only with the vicious and through the vicious, and provides communion with moral good, both in conversation and reading.

2. Solitude will usually become injurious to the health from want of employment; for it is accustomed to exercise the same disadvantageous influence even under directly opposite circumstances, that is, amidst the noise and bustle of the world. Labour is necessary to put the finer and rougher powers into action, and use up the superfluous strength, to the intent that the machinery of the mechanical part of the organism may not be put into disorder. Such a disorder so produced is undoubtedly likely to induce a morbid condition of the body, from which mental disorder may arise. Examples are not wanting of this disorder commencing in persons who have passed many months in forced inactivity, from surgical treatment, as for broken limbs, and at the same time have had relatively a too nutritious diet. For the same reason it happens, that strong persons, accustomed to a laborious and wearisome life, become insane in those prisons where no provision is made for the employment of the prisoners, and where the diet is proportionately too nutritious. The Pennsylvanian system does not by any means exclude employment of the prisoner, but rather encourages him by it, since he seeks it voluntarily and as a solace.

3. The passions must come into consideration. According to experience they act injuriously, impairing and paralyzing the nervous system of the trunk, and all the organic functions which are regulated by it: wherefore they, rather than isolation, are, even in the midst of the exciting impulses of the world, fertile sources of mental disease. Isolation is certainly advantageous: those passions which the solitary criminal has to feel are, grief for the loss of freedom, repentance for the punishment, fear and anxiety for the affliction of a monotonous future on the one hand, or of an unknown threatening future on the other. These injurious agencies (since the mental condition induced by them must be considered such to the health) another kind of punishment may certainly neutralize, by causing diversion of the mind, and by giving opportunity to indulge in vicious thoughts and feelings, as is in fact presented in the association with like-minded persons, and in the victory over the pains and penalties of the law.

The Pennsylvanian system offers as a means of neutralizing these evils only labour, and the consolation resulting from good books, and the conversation of moral and pious men, whereby appropriate views and good principles are imbibed. Need, then, the useless question be raised, which of these two methods of neutralization be the best, the most useful, most to be commended, most appropriate?

4. That by the deprivation of free and fresh air, and of exercise, solitary confinement may give rise to a morbid state of the system. By a better construction of prisons this injurious effect may be avoided. Dr. Bache, Darrach, and other Americans, demonstrate that the Pennsylvanian system of punishment does not destroy the understanding. Crawford instituted inquiries in the institution at Cherry Hill, communicated with the prisoners, and thus expressed himself as to the influence of solitude on the health, mind, and temper. The greater number of inmates had been there for more than four years: he observed nothing in their physiognomy that could lead to the opinion that their long isolation had either injured their health or weakened their intellect. Although in general serious, they were not cast down. Many spoke with an appearance of gentle mildness which he did not expect. Demetz, who also instituted careful observations of the prisoners at Cherry Hill, expressed himself still more favourably as to the operation of isolation on the mind and body. According to the reports of the directors of this institution, a residence in it is very beneficial in insanity, since, of fourteen inmates who had become insane from previous bad habits, twelve were cured—a proportion that would be thought highly satisfactory at any asylum. Dr. Bache, who was the physician to the prison at Philadelphia for many years, reports, that isolation exercises no injurious influence on the minds of the prisoners. With respect to Glasgow, all travellers confirm what Hill shows in his reports of the prison there, that solitary confinement has no bad effect on the health of the prisoners; and that, in fact, their health is much better than in the average of the class of persons from which they are taken. David, who also visited Glasgow, speaks highly of the healthy condition of the prisoners, and says, that in 1839 there was not one individual of them all affected with any traces of insanity. Brebner, who superintended the institution for thirteen years, repeated to him what Moreau-Christoph also declared, that during the whole of that time there was not a single example to show that solitary confinement had a disturbing influence on the mental state. Gosse and Coindet oppose the Pennsylvanian system specially on the grounds that they found more insane in this prison, conducted according to that system, than in those conducted according to others. This is, however, a little remarkable, when we read Crawford's report, in which he states, that he there found four insane persons only, who were evidently insane at the time of trial. David noted the highly important fact, that in the United States, where the Philadelphian system is introduced, there are no lunatic asylums; whilst these establishments exist in New York and other states, where the Auburn system has been adopted. Besides, Philadelphia has a large coloured population, and this is more liable to insanity than the white.

These indubitable facts, partly collected by Julius and Varrentrapp—these testimonies of honourable, truthful, and learned men, which sufficiently prove that the prisoners in solitary confinement are not specially affected with insanity, may at least be sufficient to show that the prejudices against the Pennsylvanian system do not rest on irrefragable facts.

The development of insanity in all prisons may be favoured by

1. An unsuitable condition of the cells. Dark, cold, damp, ill-ventilated, and new receptacles, are hurtful both to body and mind.
2. Bad furniture.
3. Scanty clothing of the prisoners.
4. Bad and insufficient food.
5. The withdrawal of all mental and bodily occupation.
6. Harsh and unsuitable labour.
7. Harsh and improper punishments.
8. The deprivation of out-door exercise.
9. The imposition of silence.
10. The withdrawal of all society, and of all means of communication with friends and relatives.
11. Improper and unkind treatment on the part of the gaolers and turnkeys.
12. Inappropriate management on the part of the chaplain.
13. Too long seclusion.

All these pernicious agencies can and must be avoided in a well-conducted cell-prison; and this being done, the keenest criticism would find nothing to object to. We have, however, no cell-prison which supplies all the requisites. Even those who are agreed as to the necessity of isolation, differ as to the means by which it may be attained; hence arises a confusion which operates injuriously. Hitherto it has been anxiously, too anxiously, attempted in the construction of cell-prisons, to obtain absolute seclusion of the prisoners. In the arrangement of the privies, windows, doors, &c., almost every possible folly has been devised; all confidence in human nature would appear to have been lost. It has been wished to exclude the prisoners hermetically from the external world in the most ingenious manner possible. Houses have in consequence been built, "which resemble bee-hives and dead-houses, from whence the prisoner never returns, but lives and dies there, far apart from the outer world." The first proposition may be true, the second is essentially false, and is only introduced to round the period, and ought not to prevent us Germans from seeking after perfection in the construction of cell-prisons, an object which certainly is within the possibility of attainment.—*Allgemeine Zeitschrift für Psychiatrie*, vol. iv. part i.

---

### INFLUENCE OF MUSIC ON THE INSANE.

A COMMUNICATION appears in the "Journal de l'Ain," of the date of July 27, ("Gazette Med. de Paris," 1846, No. 31,) respecting the very great influence that music had upon the insane in the Hôpital de la Madeleine at Bourg—the Pyrenean singers being the performers. The greatest stillness reigned in the chapel during the performance of the pieces. The females listened with astonishment; and it was only toward the close of a rattling bolero that one of them began to dance. The mountain-singers were introduced into the apartment of the noisy and maniacal females. Screams, tears, menaces, greeted the performers; when they began their melodies the patients became quiet and attentive. "*Chose etrange!*" So soon as the singers ceased to perform, the tears re-appeared; recommencing, stillness again reigned;—at the end of the performance, renewed uproar. After the singing, one of the maniacs thanked the "native singers," in a manner so correct, one might almost say so naturally (*sic*), that the performers were quite deceived as to the state of her mind.

Dr. Damerow, chief editor of the "Allgemeine Zeitschrift für Psychiatrie," and director of the institution for the insane at Halle, in commenting upon this account, expresses his surprise that the editor of the "Gazette Medicale" passes it without an editorial remark. The editor of the "Journal de l'Ain" and the Pyrenean singers knew no better than to exclaim "*Chose etrange!*" but the editor of the "Gazette Medicale" should have known that such results were not at all wonderful. Dr. Damerow observes, "Every Sunday and every week-day we can testify to the same results of music at the saying of grace before and after meals, and during divine service, which is conducted more quietly and composedly than in many a royal chapel. Is there really a difference between the quieting of the raving maniac by means of music and song, and that evinced in the same class of patients, when, in the midst of a paroxysm, saying the word 'Silence!' I place my hand on the neck to examine the carotid pulse, and in a moment they are still, and so remain as long as my hand remains, but so soon as it is removed, rave again? No! And in this respect can the theatre-going Parisians of the parterre say, as to the insane class of persons, '*Partout comme chez nous!*' The question is not as to the results; but they can be no other than those observed in the whole class of persons out of mad-houses who listen to vocal and instrumental music. With these we must and ought to be content, however varied and indefinite they may be. Man and human nature is the only just standard of judgment with reference to the influence of general impressions on the senses and minds of lunatics generally. Music is a necessary component of the life of the insane, and its action is the more healing and beneficial in proportion as the labour of it is not only *for* the sick but *from* the sick, and in proportion as the faculties of the sick are ordered and combined for a common result, each being assured of his place, his duty, and his share in the performance."

Dr. Damerow observes, that the hospital at Halle presents a remarkable example of the curative influence of music, in the case of a schoolmaster, who had suffered the highest degree of melancholia for a year and a half, who said and did little, and had only improved so much as no longer to be unclean both night and day. In consequence of a communication to the effect that formerly the patient had displayed a love and a taste for music, he was led to the piano. After some feeble touches, it might actually be seen how love and taste expanded the pinions of the soul. He not only practised more and more, and better and better, but began to compose songs for one or four voices, and practised as a "master" amongst the other patients. The musical talent was in action long before the restoration to mental and corporeal strength. As regards this, he was perfectly sound long before he was scarcely convalescent in other respects. He was dismissed cured. In this case it was not music, simply as such, which led to restoration, but the musical talent being brought into action and developed excited the latent powers of the mind, strengthened them, and kept them in action. This mental gymnastic was assisted by work in the garden during the spring, after a long tedious winter.—*Zeitschrift für Psychiatrie*, vol. iv. part ii.

---

### A BALL IN THE ASYLUM AT VIENNA.

The "Cologne Gazette" has the following:—"The experiment is now being made in the lunatic asylum at Vienna, to cheer and cure the patients by music and dancing. A ball, lately held there, was a painfully interesting scene. Beautiful well-dressed maidens, merry maskers, laughing cheerfulness, a confused crowd, in which was now and then seen an impassive countenance, features without expression, eyes glaring with fierce madness or dull idiocy, lips around which played a cold idiotic smile. A series of concerts will be held in the asylum, and the physicians promise much from the effect of music on the shattered intellects of the patients.

"A protective society will be established with the new year by the senior physician, Dr. Viszanik, for the protection of the poor and needy patients dismissed from the Vienna asylum."

---

### ON THE ADMINISTRATION OF ETHER IN THE TREATMENT OF INSANITY.

*Paris, March* 10, 1847.

Dr. Moreau (de Tours), physician of the Bicêtre, was the first to try the ether on male lunatics and epileptics, during the period of intermission, but without beneficial results. After the lapse of a few minutes, the patients inhaling it were attacked with hallucinations, delirium, &c., and instead of a soothing influence being exerted, an opposite effect resulted, and the excitement was so very great, that Dr. Moreau, according to his own confession, was not by any means encouraged to continue his researches. His colleague, Dr. Falret, instituted experiments in the Salpêtrière about the same time, and with a somewhat more fortunate result. He wished to learn, in the first place, whether insanity rendered the results different from those ordinarily consequent on the inhalation of ether, and whether it might be successfully used as a narcotic in the operations which it is sometimes necessary to practise on the insane. Falret wished also to ascertain another fact of great importance—namely, whether the changes induced in the nervous system by ether had any influence on the morbid conditions of that system, that is, in mental affections. He has already made five experiments, and as regards the first question, he can answer it partly in the affirmative. All the phenomena induced by ether in persons perfectly well, or suffering from surgical diseases, are also manifested by the insane after inhalation. But together with the ordinary phenomena, there is not the slightest change in the mental disease, but rather a manifestation of the fixed ideas and diseased condition of the patient. Paroxysms of insanity and mania return, so that Falret has renounced all hope of a beneficial use of ether in this department of medicine.

### PRIZE QUESTION OF THE UNIVERSITY OF BERLIN.

QUUM omnis medicinæ et psychologiæ ratio et disciplina e nervorum cognitione pendeat, interest scire, quantum medicorum scholæ physiologiæ compotes in hac provincia profecerint. Exponatur igitur neurologiæ historia ab Herophilo ad hoc esque tempus.

